# Studies on Aggregated Nanoparticles Steering during Deep Brain Membrane Crossing

**DOI:** 10.3390/nano11102754

**Published:** 2021-10-17

**Authors:** Ali Kafash Hoshiar, Shahriar Dadras Javan, Tuan-Anh Le, Mohammad Reza Hairi Yazdi, Jungwon Yoon

**Affiliations:** 1 School of Computer Science and Electronic Engineering, University of Essex, Colchester CO4 3SQ, UK; 2 School of Mechanical Engineering, University of Tehran, Tehran 1439955961, Iran; shahriardadras@ut.ac.ir (S.D.J.); myazdi@ut.ac.ir (M.R.H.Y.); 3 School of Integrated Technology, Gwangju Institute of Science and Technology, 123 Cheomdangwagi-ro, Buk-gu, Gwangju 61005, Korea; tuananhle@gist.ac.kr

**Keywords:** Alzheimer’s disease, hippocampus, magnetic nanoparticles, electromagnetic actuation, swarm steering, nanorobotics

## Abstract

Many central nervous system (CNS) diseases, such as Alzheimer’s disease (AD), affect the deep brain region, which hinders their effective treatment. The hippocampus, a deep brain area critical for learning and memory, is especially vulnerable to damage during early stages of AD. Magnetic drug targeting has shown high potential in delivering drugs to a targeted disease site effectively by applying a strong electromagnetic force. This study illustrates a nanotechnology-based scheme for delivering magnetic nanoparticles (MNP) to the deep brain region. First, we developed a mathematical model and a molecular dynamic simulation to analyze membrane crossing, and to study the effects of particle size, aggregation, and crossing velocities. Then, using in vitro experiments, we studied effective parameters in aggregation. We have also studied the process and environmental parameters. We have demonstrated that aggregation size can be controlled when particles are subjected to external electromagnetic fields. Our simulations and experimental studies can be used for capturing MNPs in brain, the transport of particles across the intact BBB and deep region targeting. These results are in line with previous in vivo studies and establish an effective strategy for deep brain region targeting with drug loaded MNPs through the application of an external electromagnetic field.

## 1. Introduction

Alzheimer’s disease (AD) continues to be a growing public health concern. It is estimated that there will be over 115 million new worldwide cases of AD within the next 40 years, resulting in an overwhelming health and economic burden on society [1]. The cases where AD was determined as the cause of death have increased by 68% between 2000 and 2010 [2]. Given the major public health priority of AD, the Alzheimer’s Association released the National Alzheimer’s Plan, From Act to Action, outlining a national strategy to address AD research, care, and services with the specific goal of finding effective ways to prevent and treat the disease by 2025. AD is characterized by progressive cognitive dysfunction often beginning with an early disturbance of episodic memory and ultimately leading to absolute functional impairment. The apparent pathological processes caused by AD include misprocessing of fibrillar amyloid leading to oligomerization, the deposition of amyloid plaque causing a disruption of neural network activity, a loss of synaptic function, and eventual neuronal death [3,4]. As AD patients are often resistant to pharmacotherapy, alternative therapeutic strategies are imperative.

Recently, many nanomedical studies have been focused on magnetic nanoparticles (MNPs) because MNPs possess attractive properties for potential uses in imaging, drug delivery, and theranostics [5]. The developments in MNPs for biomedical applications have significantly increased the expectations because of the versatile natural properties of MNPs for application in biological studies, such as drug delivery and imaging [6,7]. MNPs exhibit unique optical properties suitable for in vivo tracking and are capable of delivering drugs to the brain cells [8]. These advances have enabled MNPs to be safely guided and concentrated with an external magnetic field at a location of interest inside the body. Nanoparticles in drug delivery applications, including magnetic nanoparticles (Fe${}_{3}$O${}_{4}$), have been discussed in detail [9].

In magnetic drug delivery (MDD), the drug or fluorophore is conjugated to the MNPs, the particles are injected into the blood vessel and circulate throughout the vasculature network, then an external magnetic field is used to concentrate the desired compound at the desired location to produce an optimal concentration of loaded particles at that location [10,11].

Initially, the studies of MDD centered on capturing and retaining particles with stationary permanent magnets. The concentration of MNPs under a static magnetic field in simulation with a Y-shaped bifurcation was reported [12]. Molecular dynamics were used to study membrane crossing using cylindrical and spherical nanoparticles [13,14,15]. In [16], a thorough computational simulation of crossing of different shapes of nanoparticles through cell membranes was performed and the importance of initial orientation, nanoparticle volume, and anisotropy was discussed. In [17], the molecular simulation of aggregation of fullerene was performed and an optimum fullerene concentration for crossing through the skin bilayer was introduced, but a driving magnetic force was not used. The blood–brain barrier (BBB) is the border between the brain’s extracellular fluid in the central nervous system and the circulating blood flow, which controls the passage of different molecules between the blood and the brain. BBB is composed of different types of lipids including: phosphatidylcholine, phosphatidylinositol, phosphatidylethanolamine, phosphatidylserine, sphingomyelin, and cholesterol [18]. In experiments, the constant magnetic field was used for the BBB crossing, and it was observed that the particles passed through BBB by the endocytosis process; however, despite the success in BBB crossing, the sticking and aggregation were not considered [19].

Sticking refers to the sticking of nanoparticles to the blood vessels during the magnetic drug delivery [20]. This phenomenon occurs under a static magnetic field. Experimental evidence (in vitro and in vivo studies) have also showed that many particles aggregate (chain type aggregates) during magnetic guidance.

Therefore, previously in a simulation, we solved the sticking issue by intentionally changing the magnetic field direction, and the use of dynamic magnetic actuation (change in field direction) for reducing aggregation [21]. The experimental results in [22] showed that the dynamic actuation with a pulse-shaped magnetic field using permanent magnets can improve crossing of the cell barrier. To study the drug uptake, experiments with time varying dynamic magnetic actuation were performed on mice and the brain tissues were examined. In absence of the magnetic force, no evidence of nanoparticles was found in the brain, with the dynamic actuation, however, the rate of BBB crossing and drug uptake improved significantly [23].

A simulation platform for aggregated nanoparticle steering was developed [24]. The proposed platform was studied using in vitro and in vivo experiments. Models for multiple bifurcation steering have also been developed [25,26], and aggregation under a rotating magnetic field has also been studied [27]. The proposed models were centered around steering of aggregates. The effects of aggregates on BBB crossing, and parameters influencing the aggregates length have not yet been introduced.

**Figure 1:**
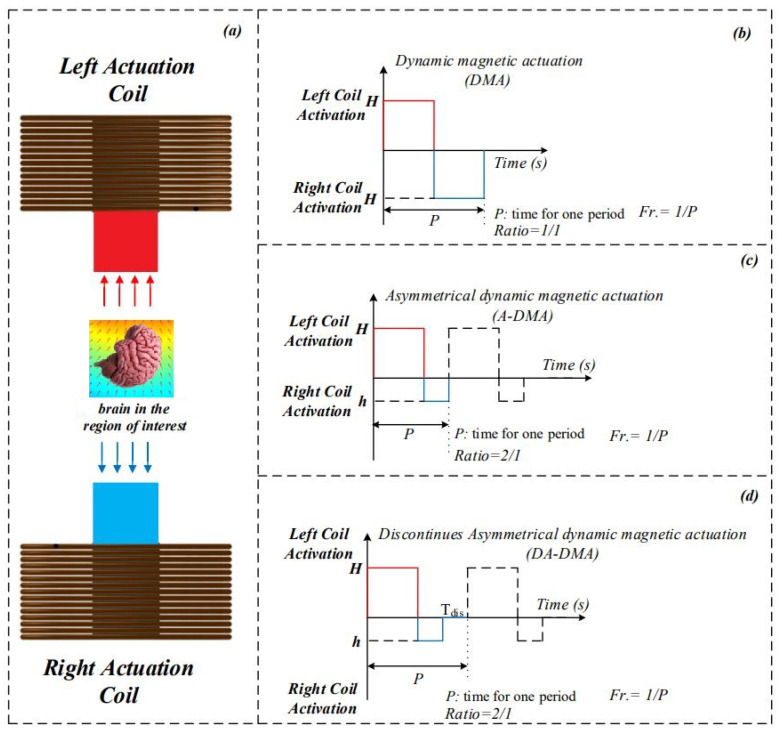
The magnetic actuation schemes; (**a**) two electromagnets are positioned on the right and left side of the brain providing a strong magnetic field; (**b**) the dynamic magnetic actuation with equal magnetic intensity (H, Fr.) [20]; (**c**) the asymmetrical dynamic magnetic actuation (A-DMA) with unequal magnetic intensity (H, h, Fr.) [28]; (**d**) the discontinuous asymmetrical dynamic magnetic actuation (DA-DMA) with unequal magnetic intensity and discontinuity (H, h, Fr., $T_{dis}$) [24].

Three types of magnetic actuation schemes have been studied. (1) a dynamic magnetic actuation (DMA Figure 1b), which has equal magnitude in both directions (H,H, Fr.) [23]; (2) an asymmetrical dynamic actuation (A-DMA Figure 1c), which has unequal magnitude (H, h, Fr.) [28]; and (3) discontinuous asymmetrical magnetic actuation (DA-DMA Figure 1d), which has unequal and discontinuous magnetic actuation (H, h, Fr., $T_{dis}$) [24]. For both A-DMA and DA-DMA, the activation time ratio between the left and right electromagnets is considered to be 2:1. The DA-DMA showed the highest performance in delivering MNPs to deep brain regions. However, the effects of aggregation on membrane crossing have not been studied. In this paper, we used molecular dynamics simulations and in vitro parametric studies of aggregation to further improve magnetic schemes for drug delivery.

In this paper, we have illustrated for the first time that the aggregated particles pass through the membrane in the simulation. We studied particle size, aggregation, and velocity of crossing in the molecular dynamics simulations. We have also experimentally studied the effective parameters on aggregation. The in situ and in vitro studies can be used to enhance the performance of actuation schemes in MDD.

## 2. Results and Discussion

### 2.1. Forces Governing Aggregation

**Figure 2:**
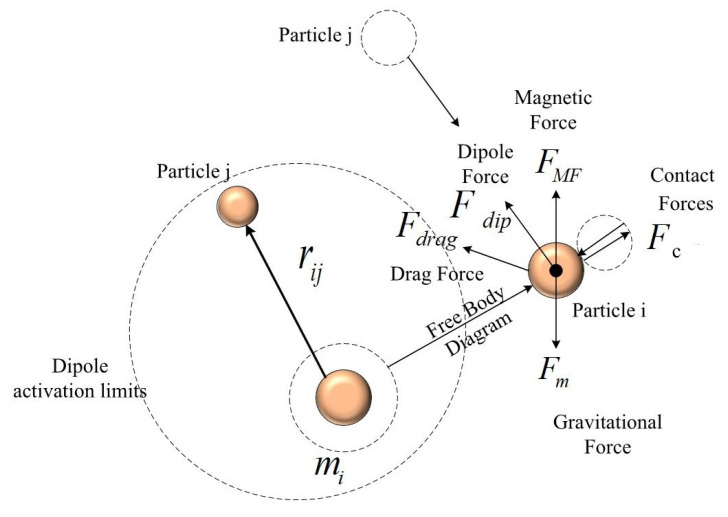
Free body diagram of the dominant forces involved.

This section presents an overview of the forces affecting aggregation. Many of the parameters presented here will be used throughout the manuscript and any change in these parameters will be described in the appropriate sections. The forces depicted in Figure 2 are considered and a Newtonian dynamic model is devised as follows:

(1) $$m_{i}\frac{dv_{pi}}{dt}=F_{MF}+F_{\mathrm{dip}}+F_{\mathrm{drag}}+F_{c}+F_{m}\;$$

where the index *i* indicates a particle *i*, $v_{pi}$ is the $i^{th}$ particle’s velocity, and $F_{MF}$, $F_{dip}$, $F_{drag}$, $F_{m}$, and $F_{c}$ are the magnetic, dipole, hydrodynamic drag, gravitational, and contact forces, respectively. The $m_{i}$ is the particle mass. To use Newtonian mechanics, particles are considered to be sufficiently large to exclude the Brownian effect [29,30], i.e., diameter >500 nm.

The electromagnetic force ($F_{MF}$) is the actuation force. Due to permeability differences, nanoparticles experience force in a magnetic field [20], which is modeled as

(2) $$F_{MF}=\frac{4}{3}\pi \mu_{1}R^{3}M_{\mathrm{sat}}.\nabla H\;$$

where the particles are considered to be uniform spheres with radius *R*. *H* is the magnetic intensity, $\mu_{1}$ is the permeability of the fluid, (∇H) is the gradient of the magnetic intensity, and $M_{sat}$ is the finite value of magnetic polarization.

$F_{dip}$ is the dipole force, which plays a major role in keeping the particles together [30]. The dipole force is given by

$$F_{dip}=\frac{3\mu_{1}m_{i}m_{j}}{4\pi r_{ij}^{4}}(r_{ij}\left(m_{i}.m_{j}\right)$$

(3) $$+m_{i}\left(r_{ij}.m_{j}\right)+m_{j}\left(r_{ij}.m_{i}\right)-5r_{ji}\left(r_{ji}.m_{i}\right)\left(r_{ji}.m_{j}\right)\;$$

where $\mu_{1}$ is the magnetic permeability of the fluid, $m_{i}$ and $m_{j}$ are the magnetic moments of the *i*th and *j*th particles, respectively, and $r_{ij}$ is the distance between particles.

The drag (hydrodynamic) force acting on a sphere is obtained using Stokes’ Law as [20]:

(4) $$F_{\mathrm{drag}}=-6\pi \eta R\left(v_{p}-v_{f}\right)\;$$

where $v_{p}$ and $v_{f}$ are the particle and fluid velocities, respectively, *R* is the particle radius, and η is the fluid viscosity.

$F_{m}$ is the gravitational force and is presented as follows:

(5) $$F_{m}=\frac{4}{3}\pi R^{3}\left(\rho_{p}-\rho_{b}\right)G\;$$

where $\rho_{p}$ and $\rho_{b}$ are the densities of the particle and fluid, and *G* is the gravity respectively.

During the guidance and particle aggregation, contact forces are generated as a result of particle—particle or particle—surface collisions. The Hertzian contact model is expressed as

(6) $$F_{c}=k\delta^{\frac{3}{2}}\overset{\mathrm{If}}{↔}d<R_{i}+R_{j}\;$$

where *d* is the particle–particle distance, $R_{i}$ and $R_{j}$ are the radii of the *i*th and *j*th particles, respectively, *k* is the spring constant, and δ is the deformation in particle.

### 2.2. Molecular Dynamics Modeling and Simulation for the Blood–Brain Barrier (BBB) Crossing

**Figure 3:**
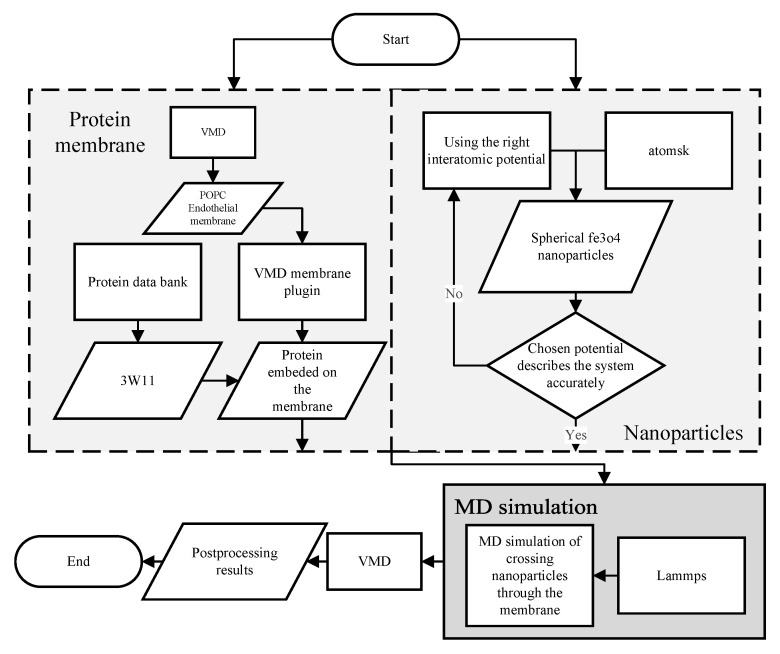
The flowchart of the MD simulation process.

Figure 3 illustrates the molecular dynamics (MD) modeling process. The membrane is designed using visual molecular dynamics (VMD) and the protein data are entered from the protein data base. In parallel, the nanoparticles are modeled using Atomsk considering the interatomic potentials. Due to the fact that choosing the wrong potential causes the nanoparticles to disintegrate, the proper interatomic potentials are used. These two models are entered into the LAMMPS MD modeling software, and the crossing forces are calculated. The membrane crossing process is visualized using the VMD software.

#### 2.2.1. Blood–Brain Barrier (BBB) Modeling

The cell membrane is modeled to study particles crossing through the BBB. The BBB consists of different types of lipids. This study is not centered on membrane construction. Due to computational limitations, the membrane is modeled using only the POPC lipid bilayer the membrane is modeled using only the POPC lipid bilayer [31,32]. POPC is a phosphatidylcholine that is composed of a diacylglycerol and a phospholipid (1-palmitoyl-2-oleoyl-sn-glycero-3-phosphocholine). A 200 A${}^{\circ }$ × 200 A${}^{\circ }$ POPC phospholipid is modeled using the VMD membrane builder software [33]. Nanoparticles crossing through the cell are mediated by receptors covering the membrane. These include the insulin receptors, which are strongly visible on the capillary endothelial cells. The insulin receptor (IR) is a transmembrane receptor activated by insulin, IGF-I, IGF-II, and belongs to the large class of tyrosine kinase receptors [34]. Protein 3W11 from the protein data bank is a suitable candidate to be used as the IR. The protein’s ectodomain, which is the part of the protein that initiates contact with surfaces, is used. We used chains A and B of this protein, which are the active parts of the ectodomain.

**Figure 4:**
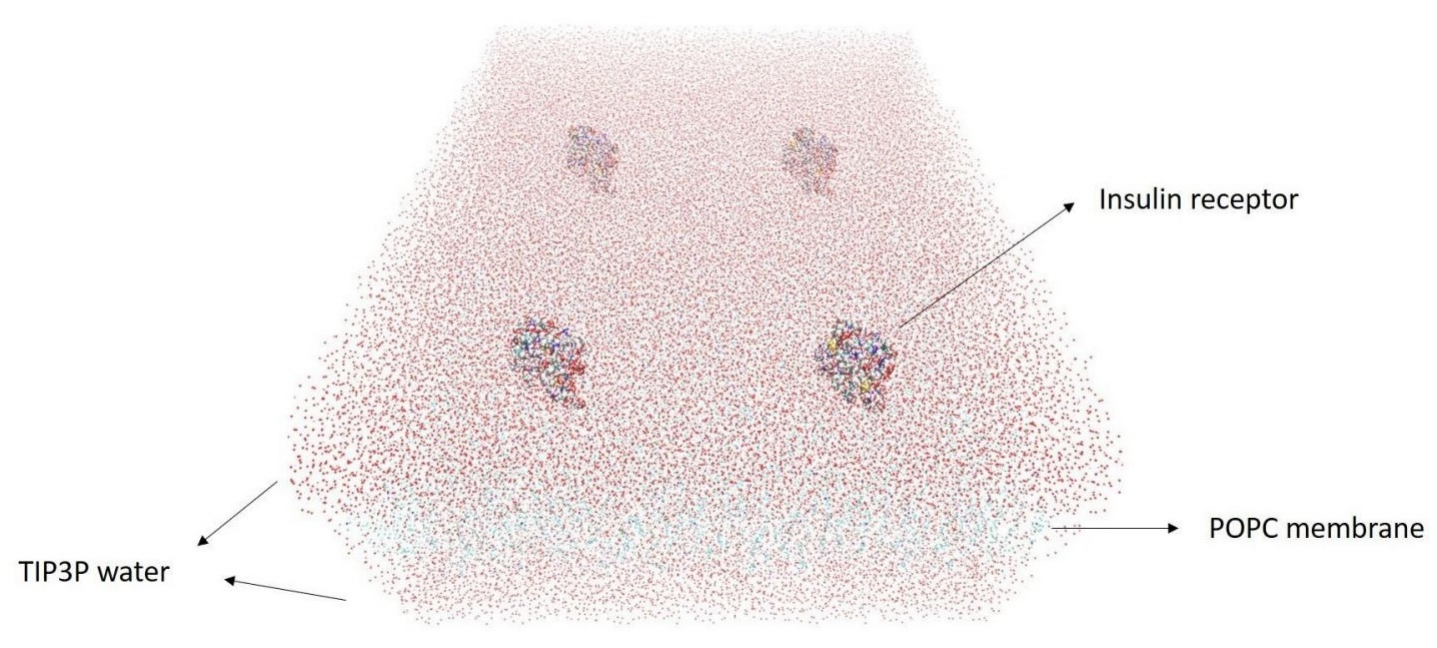
200 A${}^{\circ }$ × 200 A${}^{\circ }$ embedded membrane with four receptors and two 15 A${}^{\circ }$ layers of water.

After creating the membrane, due to the dimensions of membrane and protein we only used active parts of the protein as the receptor. We divided the membrane into four equal 100 A${}^{\circ }$ × 100 A${}^{\circ }$ sections and put a receptor on each part. For the direct interaction of the nanoparticles and the receptors, we displaced atoms of the receptor so that one whole receptor is positioned in the middle of the membrane. To neutralize the embedded membrane in terms of electric charge and in order to solvate the membrane in water, ions and TIP3P water are added to the system. We put the membrane and its receptors between two 15 A${}^{\circ }$ layers of water, the result is shown in Figure 4.

#### 2.2.2. Nanoparticles Modeling

To simulate the MD crossing of nanoparticles through the BBB, Fe${}_{3}$O${}_{4}$ spherical nanoparticles are used. The Fe${}_{3}$O${}_{4}$ crystallographic information file (CIF) is used by Atomsk software [35,36].

**Figure 5:**
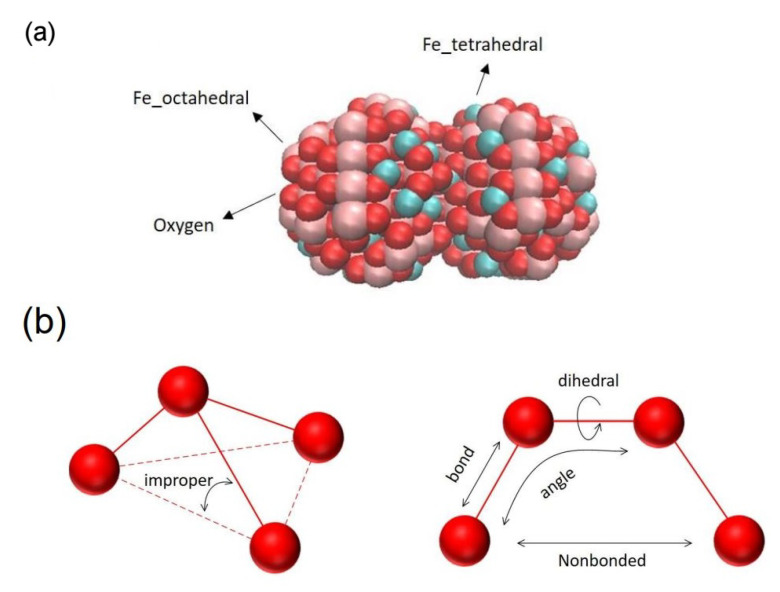
(**a**) Two 1 nm radius Fe${}_{3}$O${}_{4}$ nanoparticles attached to each other. Red atoms are oxygen, pink atoms are $Fe_{octahedral}$, and cyan atoms are $Fe_{tetrahedral}$, (**b**) schematic of the forces and atoms configuration.

To study the aggregation effect, a STL file is used in the Atomsk software and an aggregate of two spherical nanoparticles is used (Figure 5).

#### 2.2.3. Force Analysis

The molecular forces in this simulation are divided into non-bonded and bonded forces:

(7) $$F=F_{nonbonded}+F_{bonded}\;$$

The bonded forces included are categorized as (1) forces between membrane atoms, and (2) forces between nanoparticle atoms. The latter are not calculated due to the assumed rigidity of nanoparticles.

The bonded forces between the membrane atoms are divided into four main parts:

(8) $$F_{bonded}=F_{bonds}+F_{angles}+F_{dihedrals}+F_{impropers}\;$$

The forces can be modeled as derivatives of potentials. Therefore, these four main potentials are formulated as follows:

(9) $$V_{bonds}=k_{b}\times {\left(b-b_{0}\right)}^{2}\;$$

(10) $$V_{angles}=k_{\theta }\times {\left(\theta -\theta_{0}\right)}^{2}\;$$

(11) $$V_{dihedrals}=k_{\phi }\times \left(1+cos\left(n\phi -\delta \right)\right)\;$$

(12) $$V_{impropers}=k_{\omega }\times {\left(\omega -\omega_{0}\right)}^{2}\;$$

where $k_{b}$, $k_{\theta }$, $k_{\phi }$, and $k_{\omega }$ are constants [37,38], *b* is the distance between the two atoms, $b_{0}$ is the equilibrium distance, θ is the angle between three atoms, $\theta_{0}$ is the equilibrium angle, ϕ is the angle between the planes formed by the first and the last three of the four atoms, *n* is the periodicity, δ is the equilibrium angle of this potential, ω is the angle between the plane formed by the central atom and two peripheral atoms and the plane formed by the peripheral atoms, and $\omega_{0}$ is an optimal improper angle.

The non-bonded forces consist of Van der Waals and electrostatic forces. These forces can be defined as derivatives of potentials and formulated as follows:

(13) $$V_{VanderWaals}=4\epsilon \left[{\left(\sigma /r\right)}^{12}-{\left(\sigma /r\right)}^{6}\right]\;$$

(14) $$V_{electrostatic}=\left(\left(q_{1}\times q_{2}\right)\right)/\left(\left(4\times \pi \times \epsilon_{0}\times r\right)\right)\;$$

where *r* is the distance between two atoms, ϵ and σ are the depths of the potential well and the collision parameter which are determined for each atom [37,38,39], $\epsilon_{0}$ is the electric susceptibility of vacuum, and $q_{1}$ and $q_{2}$ are the charges of the two interacting atoms. The partial charges of $Fe_{3}O_{4}$ based on PBE density function method are used in this model [40]. These charges for $Fe_{3}O_{4}$ atoms are shown in Table 1, and for the membrane atoms can be extracted from [37,38].

#### 2.2.4. Molecular Dynamics Conditions

A molecular dynamics simulation was performed using a large-scale atomic/ molecular massively parallel simulator (LAMMPS) [41]. Steered molecular dynamics (SMD) were used in our simulation to pass different sizes and geometries of nanoparticles through the membrane with constant velocity. Different spring constants were used for all simulations and the average of the crossing velocity is calculated in each case. To use a common spring constant for all crossing sizes and velocities, and in order to not have excessive forces, a spring constant of 15 kcal/mole * Angstrom${}^{2}$ was used. The desired magnetic force profile can be calculated by passing the nanoparticles through the membrane with constant velocity and extracting the force applied on the nanoparticles from the membrane. By applying the same magnetic force profile on the nanoparticles, their crossing through the membrane with a desired velocity can be achieved. The simulations were carried out at 310 K. Isothermal-isobaric ensemble was used to control the simulations’ temperature and pressure using the Nose-Hoover thermostat and barostat during all simulations including equilibration. The pressure was set to 1 atm in x and y directions. Because of the empty space under the membrane in the simulation box and due to the fact that the change of size of our simulation box in the z direction was not necessary. we allowed our simulation to change size in the x and y directions, but not in the z direction. We used periodic boundary conditions in all directions. To optimize the simulations, various time steps were used for every simulation.

The Charmm27 force field was used to model the membrane atom interactions, including their bonds, angles, dihedrals, impropers, and non-bonded interactions [37,38]. The Lennard-Jones and long-range Coulombic potential was used to model the interactions between the nanoparticles and membranes and to model the non-bonded interactions between the membrane atoms [37,38,39].

**Figure 6:**
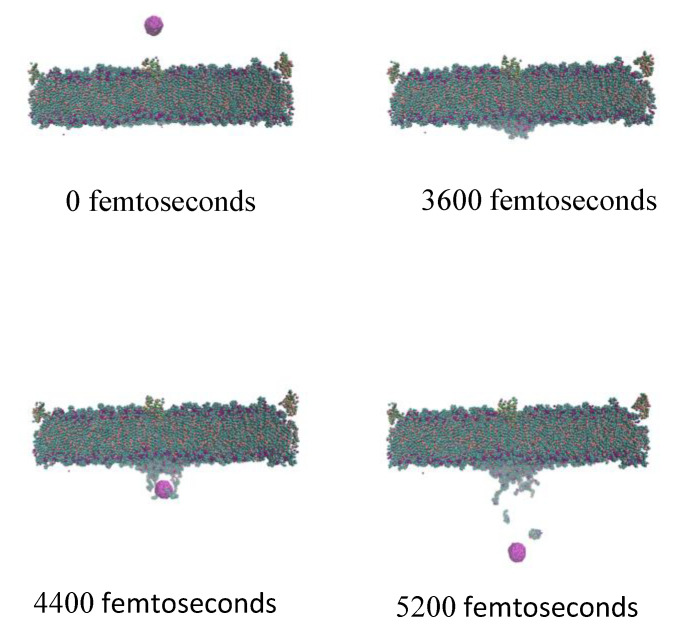
A single particle crossing the membrane in a molecular dynamic simulation.

A single Fe${}_{3}$O${}_{4}$ particle was modeled with 0.03, 0.04, and 0.05 A${}^{\circ }$/femtosecond constant velocities using the SMD method. Figure 6 shows the crossing process for the particle with 0.03 A${}^{\circ }$/femtosecond constant velocity. The applied force from the membrane on the nanoparticle depends on the crossing velocity and is calculated using this modeling approach.

All non-bonded interactions between the membrane atoms were truncated to zero beyond the center–center cutoff distance of 12 A${}^{\circ }$ with a switching distance between 10 A${}^{\circ }$ and 12 A${}^{\circ }$. A cutoff of 16.7 A${}^{\circ }$ was used for the interactions between the nanoparticles and the membrane. After energy minimization and 100 picoseconds of equilibration, the steering of nanoparticles was simulated. During equilibration based on the changes of the thermodynamics properties (pressure, temperature, potential energy, and kinematic energy), it was shown that, even in a much shorter time, the equilibration was complete. Table 2 provides the list of MD parameters used in this study for the crossing of aggregated nanoparticles. The number of atoms that leave the membrane during the crossing (lost atoms) were calculated during different types of crossings under different velocities and particle sizes. It was observed that only less than 1 percent of atoms were lost. This shows that this crossing is non-invasive and that the integrity of the membrane is maintained.

### 2.3. Effect of Velocity and Particle Aggregation on BBB Crossing

**Figure 7:**
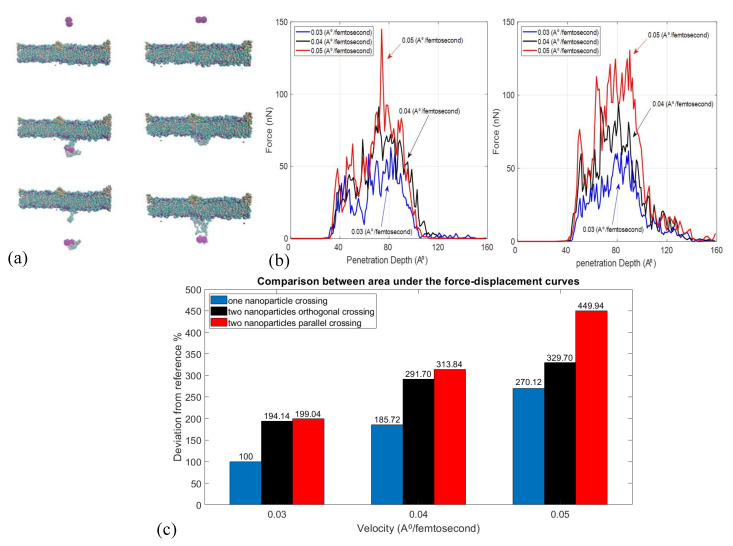
(**a**) Aggregated nanoparticles crossing the membrane in vertical and horizontal directions. (**b**) Force profiles applied by the membrane on the attached nanoparticles during the crossing with different velocities while being horizontal and vertical to the membrane. (**c**) Comparison between the required work (area under the force-displacement curve) for the crossing of a single nanoparticle with 0.03 A${}^{\circ }$/femtosecond velocity as reference and other cases.

The SMD method was used to simulate the membrane crossing (Figure 7a). As it represents the required work needed for the crossing, the area under the force-displacement curve is a suitable criterion for comparing the magnetic force for the crossing of nanoparticles through the membrane. The area under the force-displacement curve is measured to calculate the average magnetic force required for the passage. The pure force from the membrane on the nanoparticles is calculated and the velocity, particle size, and particle aggregation effects are studied. In this research, two crossing methods are studied: (1) the orthogonal crossing in which the center line of the two nanoparticles are orthogonal to the membrane surface. (2) Parallel crossing in which the center line of the two nanoparticles is parallel to the membrane surface (Figure 7b). It should be mentioned that multiple trials of molecular dynamics simulation are performed and the average results are reported. A comparison is made between the increase in the required work by an increase in volume in our study and the increase in the minimum required force for crossing a single spherical nanoparticle. An eight-fold increase in volume (radius 0.5 nm to 1 nm) of the spherical nanoparticle passing through a membrane with 0.04 A${}^{\circ }$/femtosecond constant velocity causes an 87.23% increase in the required work. A similar (88%) increase was reported in [16] for the increase of radius from 0.8 to 1.6 nm, which illustrates that both simulations follow a similar pattern despite the fact that this simulation is based on SMD and that study used dissipative particle dynamics (DPD).

The crossing of aggregates, comprised of two nanoparticles, has also been studied. As the shapes of the aggregates are not symmetric, a torque during crossing led to the rotation of aggregate. The Figure 7c shows the increase in the area under the force-displacement curve (AUFDC) for the aggregates compared to single particle under different velocities. The increase in AUFDC illustrates the importance of reducing the aggregates size to improve crossing performance. Figure 7c shows that, in the same velocity, the increase in size of the carrier significantly increases the required crossing force. Therefore, applying strategies similar to Figure 1d will help disaggregation and increase the chance of drug delivery.

### 2.4. Parameters Affecting Aggregation

**Figure 8:**
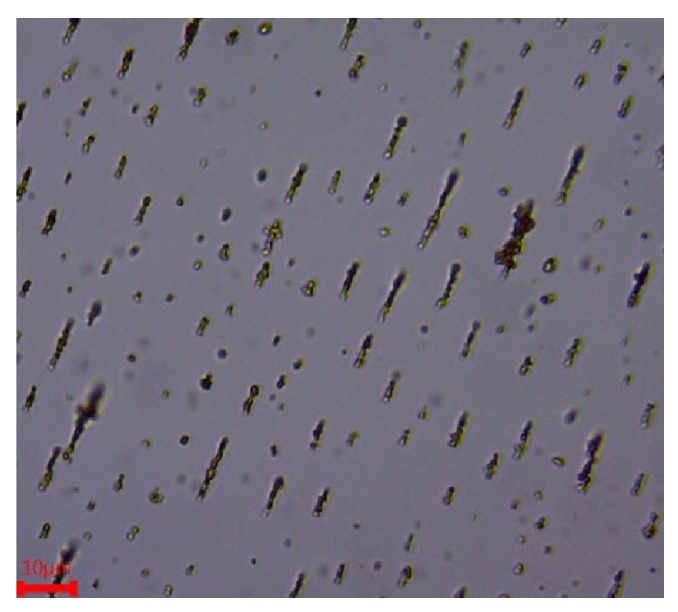
Experimental results for the aggregation of particles for 30 μL of MNPs.

The experimental results showed chain-shaped aggregates after 1 s for the 30-μL MNPs as presented in Figure 8. The magnetic force is a function of the volume of the aggregates and the gradient of the magnetic field. To study the effects of the magnetic field gradient, the actuator current was varied from 0.5 to 3 A (current field relationship is presented in Table 3) and the length of the chain-shaped aggregate was studied experimentally. In the experiments, the particle density of 30 μL of 0.5 μm MNPS (SiMAG-Silanol, Chemicell GmbH, Berlin, Germany) were used, and the results were calculated after 1 s.

The observed average aggregate size varied between 3.8 and 6.2 μm (Table 4). This can be explained by the fact that the higher magnetic gradient imposes a greater magnetic force and makes the aggregates collide faster; thus, the size of the aggregates grows in proportion with the magnetic force.

Particle size plays a similar role, when MNPs of 0.5-, 0.75-, and 1-μm diameter were exposed to the magnetic field at a current of 3 A, the initial (t = 1 s) aggregations were 2, 3.8, and 4.8 μm in length, respectively.

**Figure 9:**
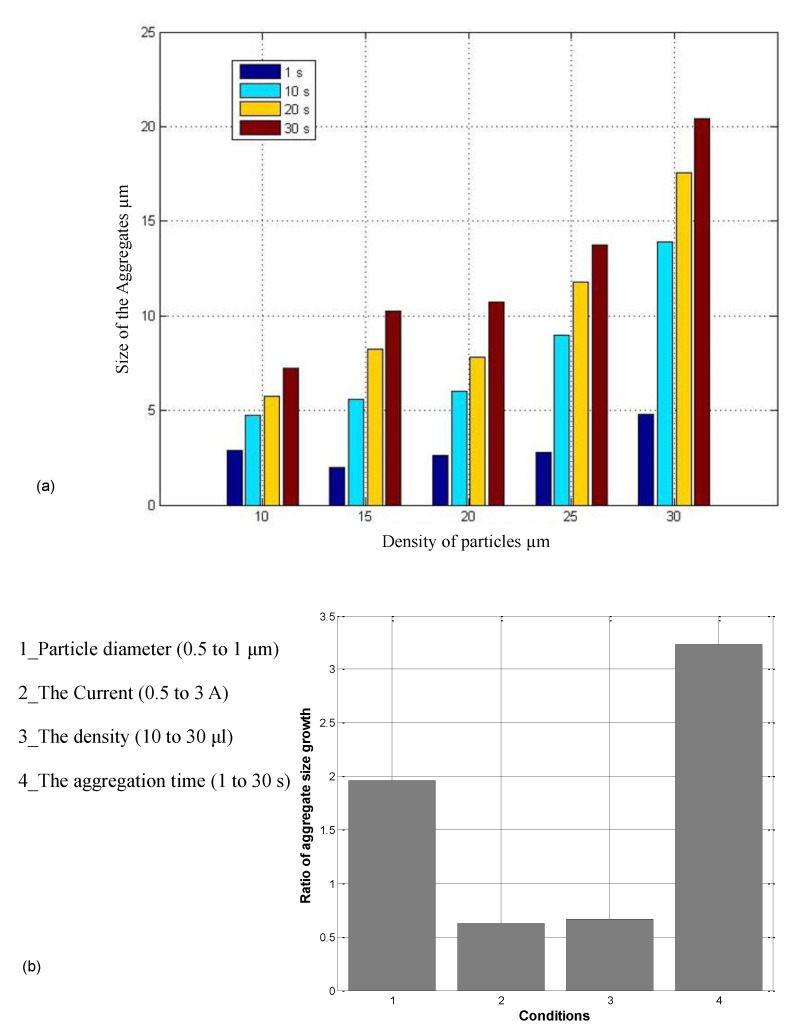
(**a**) Diagram of the aggregation time and particle density (mean value for n = 3 with less than 5% variation); (**b**) normalized aggregation rate to show the effect of particle diameter, the electromagnets’ current (representing the magnetic field), the particle density, and the aggregation time (the nominal aggregation length based on Table 5 values is 6.2 μm).

The dipole force is a function of the distance between particles distributed in the environment; the smaller the distance between particles, the larger the aggregates length will be. Given that the particles move and enter the domain of the dipole effect, it is also a function of process time. Figure 9a shows that the aggregate length grows over time for all particle distribution conditions. The ratio of the average aggregate length after 30 s with respect to the size at 1 s, for 10, 15, 20, 25, and 30 μL density of 1 μm particles is 2.5, 5.2, 4.0, 4.9, and 4.2, respectively. Moreover, the difference between the initial aggregation for particle densities of 30 and 10 μL is 1.9 μm, which shows the role of the particle distance.

Table 5 shows the effect of each parameter. Figure 9b shows the ratio of the increase in aggregate length; the experiment time had the greatest effect on particle length. After 30 s, the aggregates were three times larger than their initial size. The particle diameter was the second most effective parameter, as it increases the magnetic force, allowing the particle to move more quickly. The current and density (distance between particles) had smaller effects. Figure 9 shows that aggregates size highly depends on time of magnetic actuation. Therefore, decreasing the continues actuation time will decrease the aggregate size. Therefore, the Figure 1d can be further improved by considering these parameters and tailoring the actuation scheme based on specific nanoparticle aggregation properties (Figure 9).

### 2.5. Discussion

Over the past decades, magnetic nanoparticles (MNPs) have been the subject of increasing interest in numerous research activities, in particular for advanced medical diagnostics and therapy [42,43,44,45,46]. Due to their size, which is comparable to biological objects, MNPs pave the way for innovative medical applications by combining biology and magnetism. Usually, an MNP consists of a magnetically active core (e.g., iron oxides magnetite/maghemite) that is coated with appropriate materials (e.g., dextran, PEG, silica, styrene) to improve chemical stability under physiological conditions. In addition, suitable ligands, antibodies, or proteins are bound to the MNPs surface to enable highly selective chemical interaction with biological systems. Furthermore, the magnetism of the MNPs can be utilized for their manipulation and highly sensitive detection with the advantage of negligible magnetic background due to biological tissue. A wide variety of applications using MNPs are currently under intense investigation, such as magnetofection [47,48], molecular and cell separation [49], targeted drug delivery [50,51], hyperthermia [52], or thermoablation therapies [53], and as tools for medical imaging techniques, such as contrast agents for magnetic resonance imaging (MRI) [54] and, more recently, tracers for magnetic particle imaging (MPI) [55,56]. More specific methods are established on the labeling of MNPs with observable markers, for instance fluorophores or radionuclides that allow the quantity of nanoparticles to be observed in the form of fluorescence intensity [23,57,58].

Our previous work identified the effects of particle aggregation on nanoparticles steering [24,25], the results of MD simulation for the membrane crossing in the current work showed that in all crossing conditions (variations in the velocity and direction) the aggregates need a significantly higher crossing work compared to a single particle size under similar conditions. Therefore, reducing the aggregation while maintaining steerability can elevate the drug delivery to the brain.

Particle diameter, magnetic field intensity, density of the particles, and the exposure time are identified as effective parameters on aggregation. Figure 9a,b illustrate that, under similar conditions, the aggregation time is the most influential parameter in increasing the length of aggregates.

The best in vivo condition reported in our previous studies was the DA-DMA [23,24,28]. We observed the best results under the DA-DMA with H 6 A h 1A at a 0.144 Hz frequency (all subjects exposed to the magnetic actuation for 10 min), which induced enhanced uptake and crossing of the intact BBB and increased the FMNPs in the hippocampus. The nanoparticles coating reduces the aggregation; however, it cannot prevent it under magnetic actuation. The approach introduced in this paper can be used to further improve disaggregation by MD modeling and in vitro studies, which can lead to increased drug uptake in the deep brain region.

## 3. Experimental Section

### 3.1. Materials

For the in vitro aggregation studies, 15 to 30 μL of MNPs (SiMAG-Silanol, Chemicell GmbH, Berlin, Germany, diameter: 1 μm) were mixed with 50 μL of water and exposed to the magnetic field.

### 3.2. Experimental Setup to Study Effective Parameters on Magnetic Nanoparticles Aggregation

**Figure 10:**
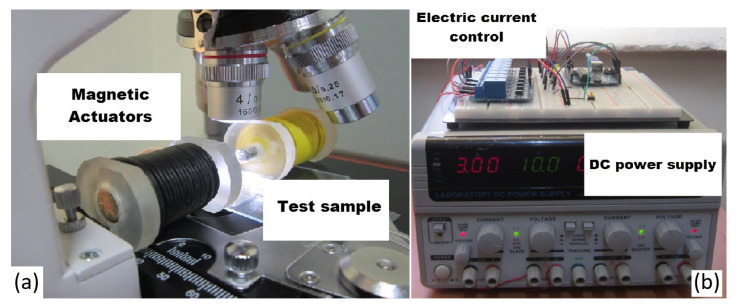
(**a**) Experimental setup used for in vitro studies of aggregation (microscope and coils), (**b**) the current control set-up.

Figure 10 shows our experimental set-up for in vitro study of aggregation effects. The experimental setup consists of three units: (1) electronic control unit (ECU); (2) two magnetic coils to generate the magnetic field; and (3) an optical microscope for monitoring.

The ECU consists of a power supply to generate 5A DC current (GW Instek GPS-4303), a microcontroller (Arduino Uno) and an 8 channel relay module (Lysignal 5V). A microscope (AmScope B120 C-E1), with resolution: 0.2 μm was used to observe aggregation. The two actuation coils create a region of interest of 10 mm.

The electromagnetic actuator comprises of two coils (25 mm diameter and 550 turns; wire diameter $d_{w}$ = 1.02 mm) with iron cores to increase the magnetic field intensity. The cores are 10 mm in length and 50 mm in diameter. The relationship between current and magnetic field generated in the region of interest is given in Table 3 in mT.

## 4. Conclusions

Here, for the first time, we used an MD model and showed the effects of aggregation, particle size, and velocity on membrane crossing. We also experimentally studied effective parameters on aggregation to design magnetic actuation schemes while considering the aggregation effects. These actuation schemes can optimize drug targeting and reach the deeper region of the brain (hippocampus) without affecting the BBB integrity.

Our previous works showed that the magnetic actuation schemes had significant improvement in nanoparticle uptake compared to the control group. The nanoparticles uptake into the brain was significantly higher under DA-DMA compared to the other schemes. In the DA-DMA, the particles are disaggregated due to the discontinuity in the actuation. Based on MD simulation and in vitro studies proposed here, the DA-DMA can minimize aggregation. The future works can benefit from this modeling and experimental approach. Similar steering algorithm without loss of generality can also be implemented for the Multi-coil systems.

In conclusion, these magnetic nanocarriers and our actuation scheme possess great potential for delivering the particles to the hippocampus. Similarly configured, but drug-containing magnetic nanoparticles can be utilized to reach the deeper regions of the brain in order to treat various CNS diseases.

## Figures and Tables

**Table 1:** Units for magnetic properties.

Atom	q (|e|)
$Fe_{tetrahedral}$	+1.68
$Fe_{octahedral}$	+1.60
O	−1.22

**Table 2:** MD parameters used in this study.

Parameter	Value/Name
Membrane type	POPC with two 15 nm layers of water
Membrane number of atoms (without waters)	152,874
Membrane number of atoms (with waters)	382,098
Nanoparticle type	Fe${}_{3}$O${}_{4}$
Temperature	310 K
Relaxation time	100,000 femtoseconds
Simulation box size	200 A${}^{\circ }$ × 200 A${}^{\circ }$ × 370 A${}^{\circ }$

**Table 3:** Magnetic fields generated (mT) in the region of interest for different currents.

			Distance		
Current	1 mm	2.5 mm	5 mm	7.5 mm	10 mm
1 Amp.	17	8	4.5	2.3	1.6
2 Amp.	28	13	10	5.5	3.5
3 Amp.	43	25.5	13	7	4.5
4 Amp.	53	25	12.5	7.8	5.1
5 Amp.	58	34.5	16	11	7.2

**Table 4:** Effects of electromagnetic actuation on aggregate size for 0.5 μm MNPS, with 30 μL densities after 1 s.

Current (Amp)	0.5	1	1.5	2	2.5	3
Aggregates size (μm)	3.8	4.4	4.5	4.8	5.4	6.2

**Table 5:** Nominal values and the ranges of change in the effective parameters.

	Diameter (μm)	Time (s)	Current (A)	Density (μL)
Nominal	0.5	1	3	30
Range	0.5 to 1	1 to 30	0.5 to 3	10 to 30
